# Less translational control, more memory

**DOI:** 10.7554/eLife.00895

**Published:** 2013-05-28

**Authors:** Graham D Pavitt

**Affiliations:** 1 **Graham D Pavitt** is at the Faculty of Life Sciences, University of Manchester, Manchester, United Kingdomgraham.pavitt@manchester.ac.uk

**Keywords:** eIF2, eIF2B, ATF4, integrated stress response, unfolded protein response, memory consolidation, Human, Mouse, Rat

## Abstract

A small molecule can enhance the memories of rats and mice by blocking the integrated stress response in these animals.

**Related research article** Sidrauski C, Acosta-Alvear D, Khoutorsky A, Vedantham P, Hearn BR, Li H, Gamache K, Gallagher C, Ang KK-H, Wilson C, Okreglak V, Ashkenazi A, Hann B, Nader K, Arkin MR, Renslo AR, Sonenberg N, Walter P. 2013. Pharmacological brake-release of mRNA translation enhances cognitive memory. *eLife* **2**:e00498. doi: 10.7554/eLife.00498

**Image** Immunofluorescence images of cells treated with ISRIB, a small molecule that enhances the memory of mice and rats by blocking a cellular pathway that controls protein synthesis

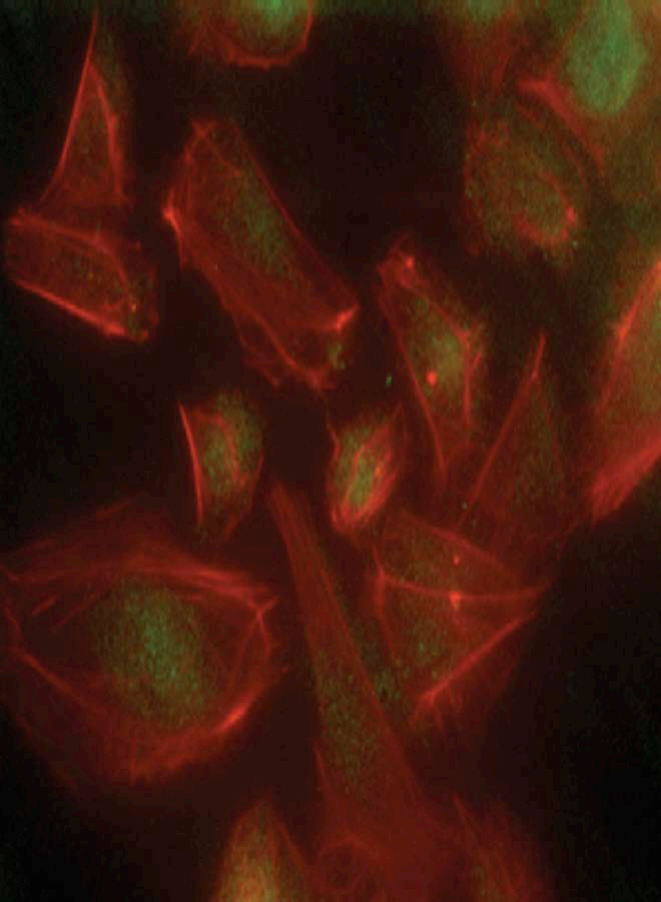

In the classic children’s book *Mrs Frisby and the rats of NIMH*, written by Robert C. O’Brien in 1971, Mrs Frisby is a field mouse aided by super-intelligent rats. In the story readers learn that scientists experimenting at the National Institute of Mental Health (NIMH) have injected rats and mice with chemicals to improve their memories. The chemicals work so well that the animals learn how to read and, ultimately, escape from the laboratory… Now Peter Walter of the University of California at San Francisco and colleagues, including Carmela Sidrauski of UCSF as first author, report that they have identified a small molecule that enhances the memory of mice and rats by blocking a highly-conserved pathway for the control of protein synthesis (Sidrauski et al., 2013).

The new study begins with research into a cellular pathway called the unfolded protein response (Figure 1; Walter and Ron, 2011). Many proteins are synthesised and folded in the endoplasmic reticulum, and when this organelle is under stress (that is, when it is unable to cope with its workload), three sensors (called PERK, IRE1 and ATF6) send signals to the rest of the cell to perform two tasks: to coordinate various ways of reducing the expression of genes, and to increase the protein folding capacity of the cell to meet demand. This two-pronged response involves changes at both the transcriptional level (in the cell nucleus) and the translational level (in the cytoplasm).

**Figure 1.**
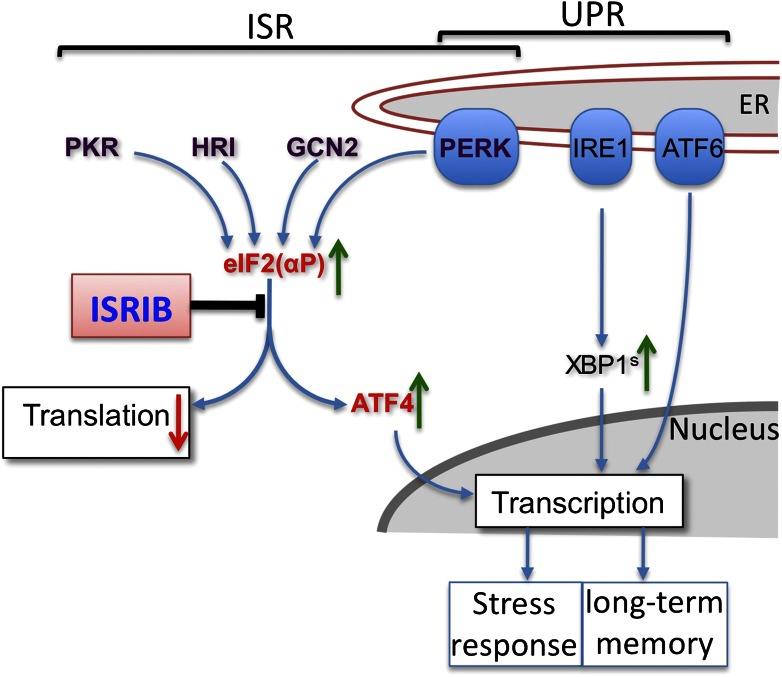
Cells respond to stress through the unfolded protein response (UPR; right)—which is caused by high levels of unfolded or misfolded proteins—and the integrated stress response (ISR; left)—which has multiple activators. ISRIB is a small molecule that acts at the intersection of these two responses. UPR stress sensors (blue ovals) localized at the endoplasmic reticulum (ER) and ISR kinases (purple text) receive stress signals (not shown) and relay these (blue arrows) to the cytoplasm and nucleus to reduce the expression of genes. The PERK pathway is involved in both responses; the signal from IRE1 is relayed via a protein called XBP1. ISRIB acts downstream of the phosphorylation of eIF2 (eIF2(αP)) and upstream of the activation of ATF4 (green arrow) and the repression of bulk protein synthesis (red down arrow). Sidrauski et al. show that ISRIB also enhances memory in rats and mice.

Walter and colleagues—who are based at UCSF, McGill University and Genentech—set out to identify an inhibitor molecule that would block the PERK arm of the unfolded protein response. PERK works by phosphorylating a protein called eIF2 that is needed to start the translation of messenger RNA into strings of amino acids, which fold to form proteins. PERK is one of four protein kinases that phosphorylate eIF2 in mammals in response to different signals. eIF2 contains three subunits, and the four kinases all phosphorylate the same serine 51 location within the alpha subunit (Figure 1). Once phosphorylated, eIF2 forms a complex with a second factor called eIF2B, and this complex reduces the overall levels of protein synthesis within the cells (Jackson et al., 2010). Paradoxically, these stress-induced signalling events also enhance the translation of the messenger RNAs for some proteins, including a transcription factor called ATF4 that modulates the expression of various genes, to ameliorate the perceived stress. The actions of these four kinases in response to stress—phosphorylation of eIF2 and increased translation of ATF4—is termed the integrated stress response (ISR).

Sidrauski et al. screened compound libraries to identify molecules that did not induce ATF4 expression under conditions of ER stress. They found a small molecule called ISRIB (short for ISR InhiBitor) that attenuates ATF4 induction without altering the IRE1 or ATF6 responses. Subsequent tests convincingly showed that this molecule does not prevent the activation of PERK or the phosphorylation of eIF2α. This means that ISRIB—unlike GSK2606414, a recently identified small molecule that inhibits PERK (Axten et al., 2012)—is not an eIF2α kinase inhibitor. The UCSF-McGill-Genentech team also showed that ISRIB could prevent ATF4 expression following activation of two of the other kinases in the integrated stress response (GCN2 and HRI). Equally importantly, ISRIB also prevented the reduction in overall protein synthesis that is normally observed within cells when eIF2 phosphorylation is high following integrated stress response kinase activation. This shows that ISRIB is acting on protein synthesis control rather than an ATF4-specific response.

Having shown that ISRIB can inhibit one of the most conserved translational control pathways in eukaryotic cells, a remaining challenge is to identify the molecule that it targets. The prime candidate is eIF2B, which is normally inhibited by eIF2α phosphorylation, and Sidrauski et al. suggest that ISRIB might either boost eIF2B activity or reduce its sensitivity to eIF2 phosphorylation. Both are plausible ideas, but other explanations cannot yet be excluded. Genetic studies using yeast have identified many mutations in eIF2 and eIF2B that permit eIF2 phosphorylation but prevent the downstream signalling responses (Pavitt, 2005). The impact of ISRIB on the integrated stress response appears to mimic the effects of these mutations.

The control of protein synthesis is important in many contexts, including the establishment of long-term memories. Experiments in which the genes involved in the integrated stress response are manipulated revealed that the modulation of this pathway influences learning and memory. For example forebrain-specific knock-out of PERK function in mice reduces both the phosphorylation of eIF2 and the expression of ATF4, leading to impaired behavioural flexibility (Trinh et al., 2012). By contrast, the induction of PKR—one of the four kinases in the integrated stress response—in neurons of the hippocampus increases eIF2α phosphorylation and ATF4 expression, but again leads to impaired memory (Jiang et al, 2010).

Sidrauski et al. now show that administering ISRIB to mice and rats results in the enhancement of their spatial and fear-conditioning memories. These results are consistent with previous work by Nahum Sonenberg of McGill University, who is part of the UCSF-McGill-Genentech collaboration: Sonenberg and co-workers replaced the serine 51 residue that the kinases bind to with an alanine residue within one of the two eIF2α gene copies present in every cell in a mouse model, and showed that eIF2α phosphorylation signalling was reduced, but not eliminated, and that memory was enhanced in a range of tests (Costa-Mattioli et al., 2007).

Taken together with other studies, it is clear that signalling through eIF2α is finely balanced, and that the chronic loss or stimulation of a single pathway can have highly deleterious effects. This is also observed in human disease. Mutations in the gene that codes for PERK cause Wolcott-Rallison Syndrome, a recessive and severe form of diabetes with bone abnormalities and mental retardation, and mutations in the genes that code for eIF2B cause VWM disease (leukoencephalopathy with vanishing white matter), a disorder that affects the formation of white matter within the brain (Pavitt, 2005). It has also been reported that brain samples from patients diagnosed with schizophrenia had lower levels of PERK and ATF4 than normal samples (Trinh et al., 2012).

It is tempting to speculate that ISRIB, or a derivative of this compound, may have beneficial effects for patients with diseases where memory is impaired. However eIF2α phosphorylation affects multiple tissues and pathways, not just the brain: Wolcott-Rallison Syndrome, for example, also affects the bones, kidney and liver, and eIF2 phosphorylation is also known to affect metabolism (Baird and Wek, 2012). Reduced eIF2α signalling is also known to increase the sensitivity of cells to viral infection (Elsby et al., 2011), so it seems possible that ISRIB could similarly reduce cellular resistance to viral infection. When ISRIB was combined with acute stress on the endoplasmic reticulum, the rate of cell death increased (Sidrauski et al. 2013).

It is clear that ISRIB will be an excellent experimental tool to help provide deeper understanding of the role of the integrated stress response, but it is likely that further advances will be needed to develop ISRIB, or a derivative, into a therapeutic that could treat memory disorders without unwanted side effects.
